# Co-existent breast malignancy and contralateral primary axillary tuberculosis

**DOI:** 10.1259/bjrcr.20210071

**Published:** 2022-01-28

**Authors:** Sam Dluzewski, Adam Brown, Besma Musaddaq, Rosalyn KF Hogben, Anmol Malhotra

**Affiliations:** 1 Department of Radiology, Royal Free Hospital, Pond St, London, UK; 2 Department of Surgery, Imperial College Healthcare NHS Trust, London, United Kingdom

## Abstract

Breast tuberculosis is an extremely rare entity representing less than 0.1% of all breast disease in developed countries. Tuberculous infections within the United Kingdom have seen a steady decline with the highest rates present within North West London where infection rates reach 24.8 per 100,000.

The presentation can mimic malignancy and lymphatic involvement of the breast both clinically and mammographically, with nodules within the upper outer quadrant, making accurate diagnosis challenging. Approximately, 30% of breast TB cases present with axillary lymphadenopathy and a recent case series review of approximately 44 cases in London found that the most common presenting feature was a solitary breast lump in 87% of cases.^4^

We present a case of a patient presenting with primary malignancy and contralateral nodal disease highly suspicious for breast malignancy. Subsequent investigation led to the identification of synchronous localized cancer and tuberculous lymphadenitis.

Synchronous presentation is uncommon and recognition and differentiation is vital as axillary lymph node metastasis is the most important factor in the staging of breast carcinoma and determining the subsequent oncological and surgical management.

## Clinical presentation

A 60-year-old white British female patient presented to breast clinic with a palpable lump within the upper outer quadrant of the left breast. The patient has no significant past medical history. Clinical examination confirmed the presence of the lesion within the upper outer quadrant of the left breast and further enlarged left sided axillary lymph nodes. The patient was referred for immediate completion of triple assessment which consists of clinical examination, mammography and ultrasonography. All clinical markers at presentation including vital signs, blood panel including inflammatory markers and urinalysis were normal. The patient had not received a recent intramuscular COVID vaccination at the time of presentation.

## Differential diagnosis

The leading diagnosis for a new finding of breast lesion with enlarged axillary nodes is metastatic primary breast malignancy. In the context of contralateral nodal disease and in the absence of ipsilateral nodal disease contralateral nodal metastases or occult ipsilateral malignancy must be considered and investigated. In the current TNM staging system, contralateral lymphadenopathy would represent metastatic malignancy rather than regional disease causing a different stage and treatment approach.

Infective and inflammatory causes of lymphadenitis must also be considered. The COVID-19 pandemic and subsequent mass vaccination program mean that intramuscular vaccination administration is an important consideration.^1^

## Investigations/Imaging findings

**Figure 1.**
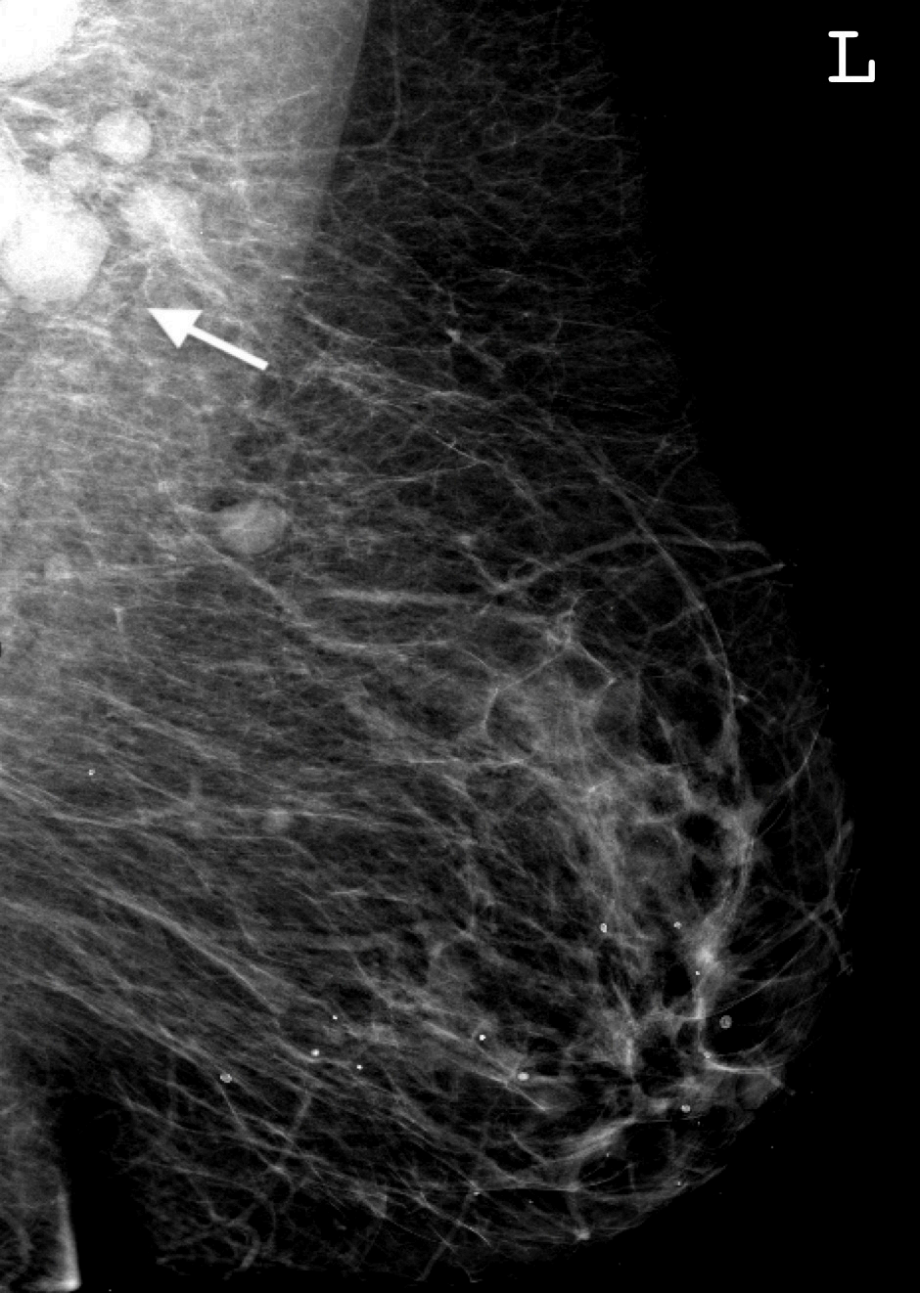
Mediolateral oblique view mammogram demonstrating axillary lymphadenopathy.

**Figure 2.**
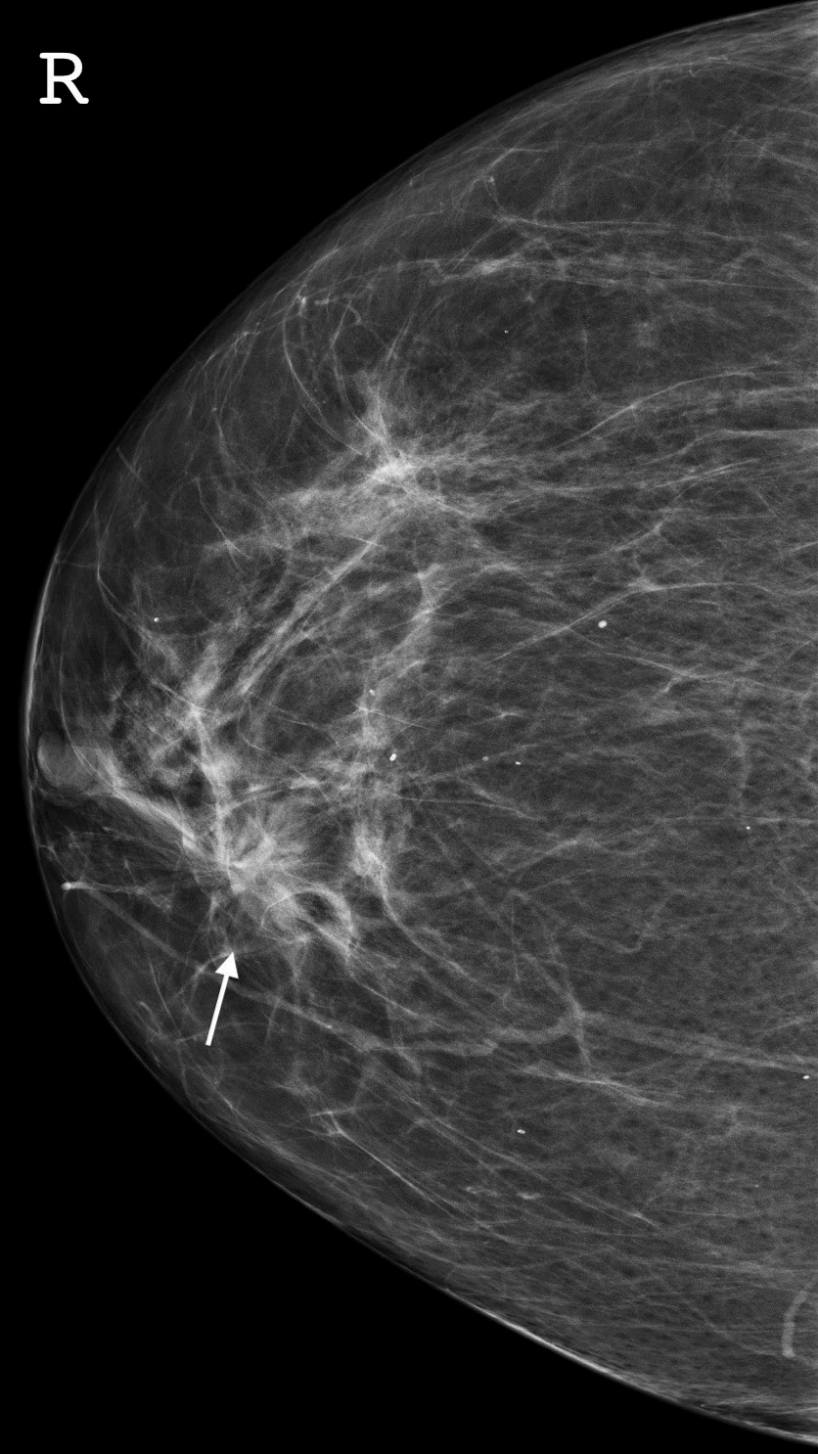
Craniocaudal view mammogram with spiculated architectural distortion.

Mammography identified large unilateral lymph nodes within the left axilla measuring up to 22 mm in long axis (Figure 1). Incidental contralateral spiculated architectural distortion and calcification was also identified within the upper inner quadrant of the right breast (Figure 2). Supplemental tomosynthesis confirmed the presence of a clinically occult right-sided breast lesion.

**Figure 3.**
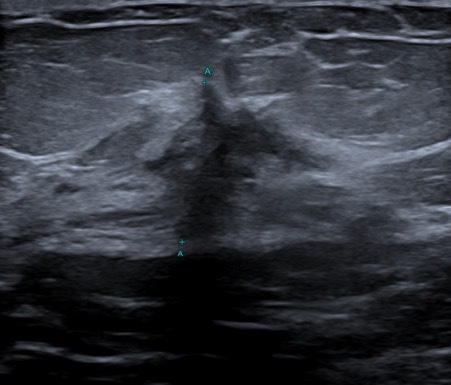
Right breast ultrasound of the spiculated lesion identified on the mammogram.

**Figure 4.**
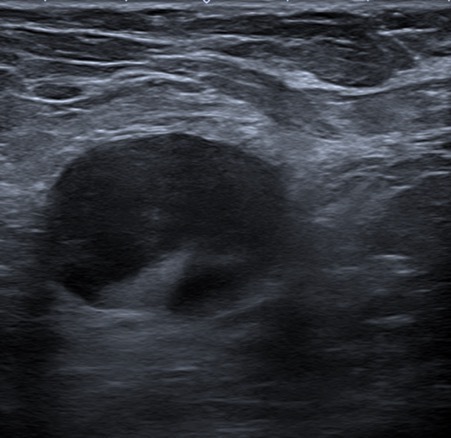
Ultrasound of the left breast with enlarged axillary lymph nodes.

Ultrasound interrogation of the right breast confirmed a highly suspicious 15 mm ill-defined mass with morphologically normal right axillary lymph nodes (Figure 3). Ultrasound of the left breast identified enlarged morphologically abnormal axillary lymph nodes (Figure 4) and an additional enlarged intramammary lymph node. The largest lymph node measured in excess of 30 mm with a cortical thickness greater than 17 mm. There was no sonographic evidence of calcification or necrosis within the lymph nodes and the lymph node was not hyperemic on doppler imaging. Comparison with prior thorax CT imaging, performed in the private sector to investigate respiratory symptoms, 6 months earlier showed normal thoracic appearances with no axillary lymphadenopathy.

Core biopsies of the right-sided upper inner quadrant lesion and the left axillary lymph nodes were performed and sent to histopathology with the initial differential diagnosis including an occult left-sided lesion with axillary metastases or rare contralateral axillary lymph node metastases.^2^

**Figure 5.**
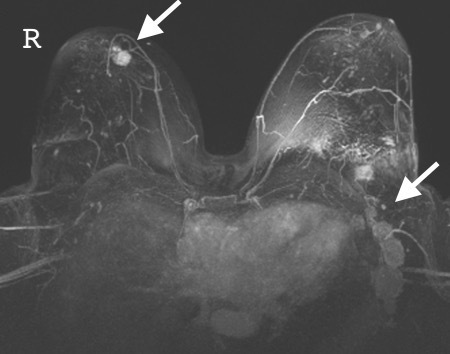
MRI with enhancing primary right breast lesion and axillary lymphadenopathy.

**Figure 6.**
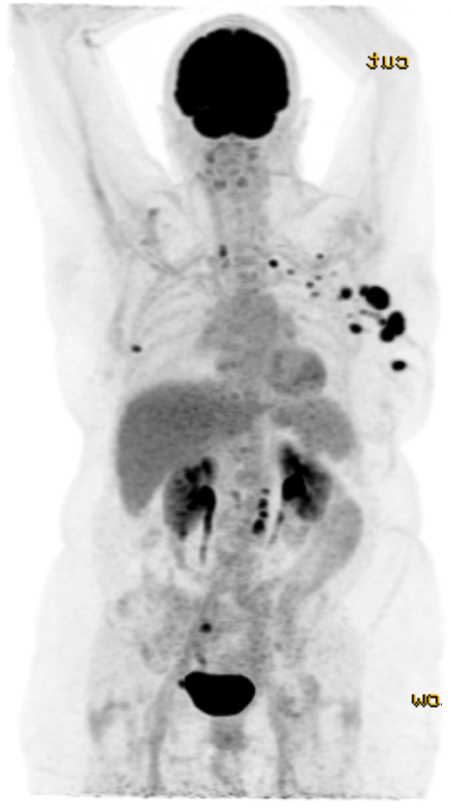
FDG PET CT demonstrating intensely FDG avid right-sided breast lesion with further intensely metabolically active left-sided axillary, internal mammary and mediastinal lymph nodes. FDG, fludeoxyglucose; PET, positron emission tomography.

**Figure 7.**
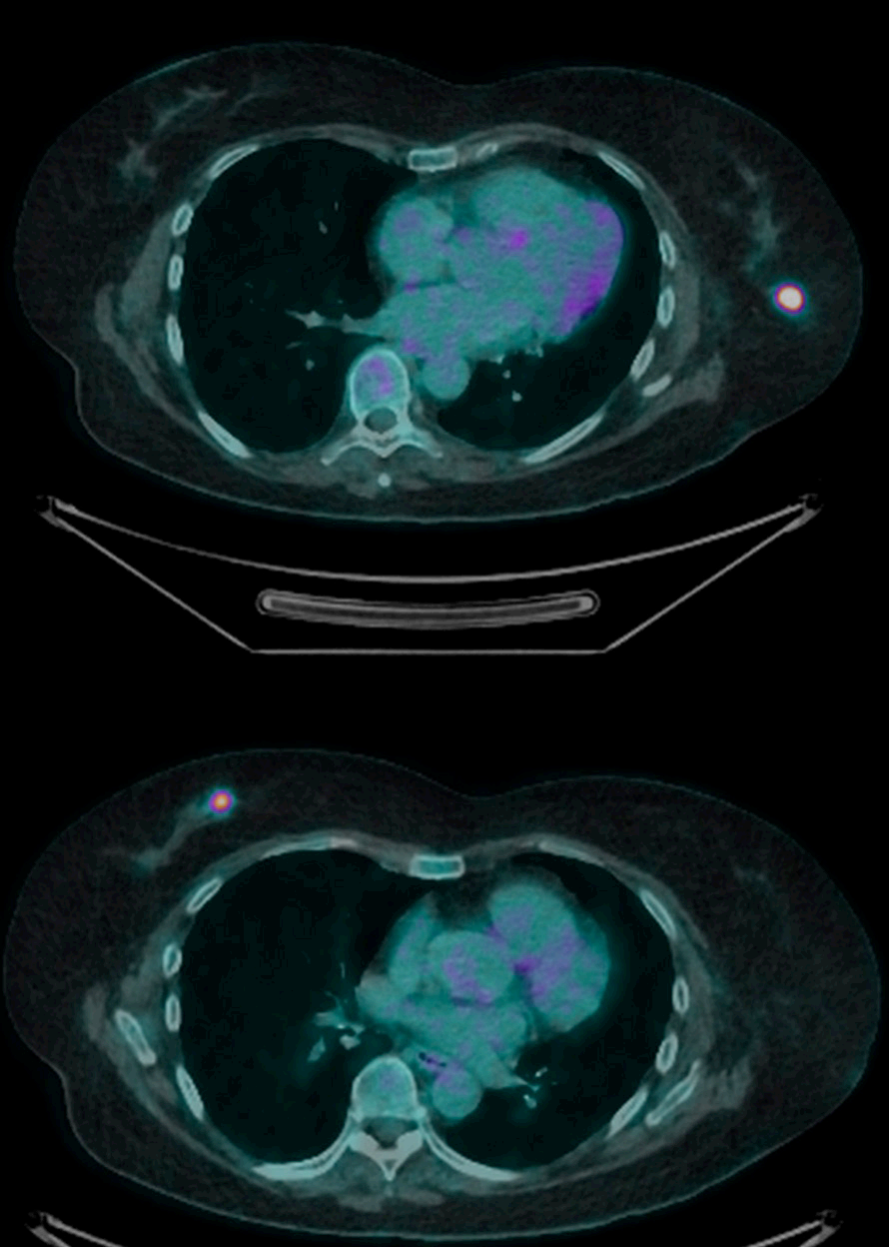
FDG PET CT with avid right-sided breast lesion (7a) and left-sided intramammary lymph node (7b). FDG, fludeoxyglucose; PET, positron emission tomography.

An MRI of both breasts was subsequently performed to assess the degree of disease infiltration, to identify a possible mammographically and sonographically occult left-sided lesion and to aid surgical planning (Figure 5). The study confirmed multiple enlarged highly abnormal left axillary lymph nodes including a left breast intramammary lymph node with evidence of recent biopsy. Further enlarged axillary nodes at levels I, II and III and supraclavicular nodes measuring up to 3 cm in diameter were also identified Figure 6. Axial post-contrast *T*_1_ weighted subtracted MR demonstrated a poorly defined enhancing lesion, with malignant Type 3 enhancement pattern within the upper inner quadrant of the right breast corresponding to the architectural distortion present on the mammogram. No further abnormality identified and no left-sided occult malignancy was uncovered. Specifically, no imaging features of tubercular mastitis were demonstrated. An ^18^F-FDG PET/CT was performed to further aid accurate disease staging. This identified an intensely FDG avid right-sided breast lesion (Figure 7a) with further intensely metabolically active left-sided axillary, intramammary (Figure 7b) and mediastinal lymph nodes but no evidence of solid organ metastases.

## Treatment

**Figure 8.**
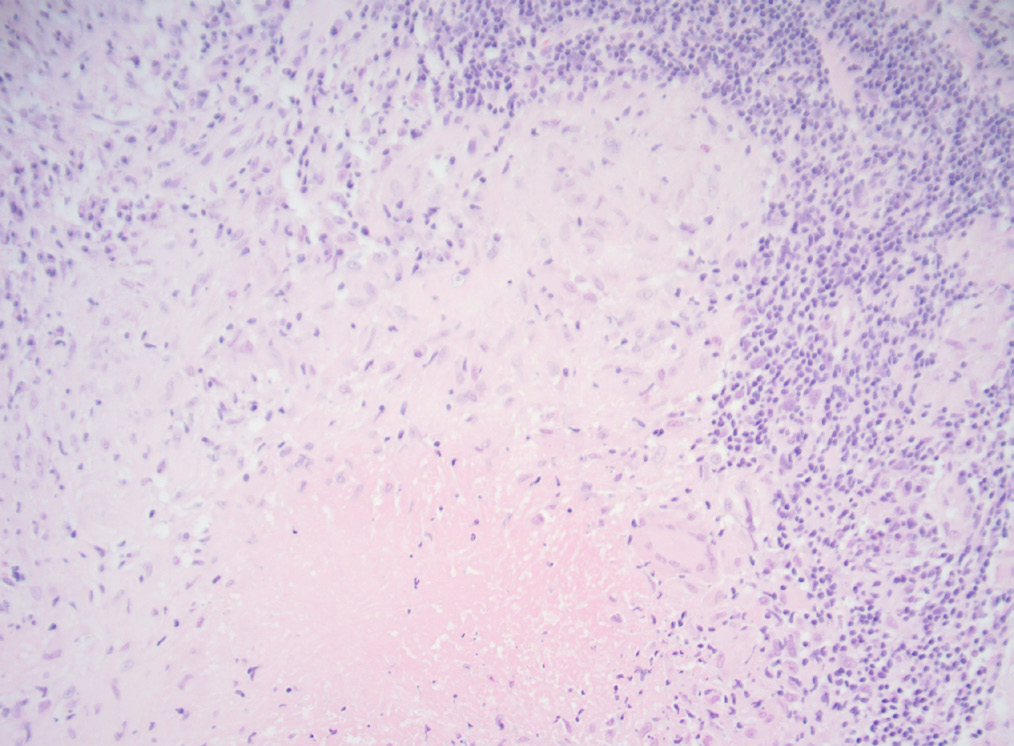
Left axillary lymph Node: There is granulomatous inflammation with central caseous necrosis and associated Langerhans giant cell x200.

The case was discussed at the multidisciplinary meeting where the histopathology confirmed the right upper inner quadrant lesion as a Grade 2 invasive lobular carcinoma within the right breast. The left-sided nodal biopsies demonstrated granulomatous inflammation with central caseous necrosis and an associated Langerhans giant cell (Figure 8) in keeping with tuberculous adenitis. No evidence of contralateral nodal metastases was found. The subsequent management was altered accordingly.

The breast malignancy was localized pre-operatively with a merit savi scout and a wide local excision was performed. The patient was treated with antitubercular drugs for 6 months with an uncomplicated post-treatment course.

### Outcome, follow-up and discussion

Breast tuberculosis represents less than 0.1% of all breast disease in developed countries^3^ with a steady decline in infections within the United Kingdom. The highest rates present within North West London where infection rates reach 24.8 per 100,000^4^.

Malignancy complicated by tuberculosis is commonly reported in literature, particularly with hematological malignancies, but is relatively rare in patients with breast cancer particularly outside of endemic areas.^5^ Breast tuberculosis is a rare disease with an incidence of less than 5% even in endemic regions and is often associated with co-existent immunological dysfunction. In a survey of British-Asian females, breast TB comprised 2.3% of all notified cases of tuberculosis.^6^ Cases of concomitant breast malignancy and tuberculosis are usually identified with ipsilateral axillary lymphadenopathy.^7^ All of the imaging modalities were utilized in this case and none served as a discriminator against metastatic spread.

Ultrasound is the primary nonsurgical method for the evaluation of axillary nodes and in breast cancer axillary lymph nodes can be classified according to cortical morphologic features.^8^ The cortex should appear hypoechoic and thin with a measurement of less than 2.3 mm as normal. Additionally, the echogenic hilum should constitute the majority of the node. In our patient, the axillary nodal disease demonstrated extensive cortical thickening measuring up to 16 mm with one of the intramammary lymph nodes demonstrating complete hilar effacement consistent with a highly suspicious Type 6 lymph node.

The differential for unilateral axillary lymphadenopathy is wide and includes malignancy as well as a range of benign causes such as infection, inflammation or recent vaccination, and therefore prompt investigation is warranted. The recent COVID-19 pandemic has caused similar diagnostic difficulties in oncological patients. Widespread vaccination has led to diagnostic dilemmas that were previously much less common.^1^ In patients with a history of recent COVID-19 vaccination, short-term imaging follow-up should be considered in an attempt to avoid unnecessary biopsy.

An important risk factor for contracting TB in the context of breast clinic is that of lactating breast feeding patients. This is believed due to breast hyperemia and subsequent hematogenous spread or transmission through baby’s saliva^9^ and these patients should be treated with a higher index of suspicion particularly in areas of high prevalence. The gold-standard for the diagnosis is detection of *M. tuberculosis* by Ziehl‐Neelsen staining or by culture. Histopathology can identify granulomatous inflammation with central caseous necrosis and Langerhans giant cells as was found in our case. The clinical presentation, however, is highly variable and can range from localized mass lesions to diffuse inflammatory involvement formally classified as nodular, diffuse and sclerosing subtypes. The nodular type can mimic breast carcinoma with an irregular fixed lump that can involve the chest wall.^10^

## Learning points

Non-metastatic causes for axillary lymphadenopathy are a highly important consideration in patients with primary breast lesions in the era of the COVID pandemic.^11^ The protean presentation of TB leads to significant diagnostic delay compounded by multiple possible differential diagnoses. This case is an important example which illustrates the limitations of nodal interpretation on multimodality imaging and the importance of corroborating histopathology. The simultaneous occurrence of tuberculosis and carcinoma can create a dilemma in the diagnosis and treatment and recognition of alternative causes of axillary lymphadenopathy is important, particularly in this unprecedented era of mass vaccination.
